# Antiproliferative effects of lanreotide autogel in patients with progressive, well-differentiated neuroendocrine tumours: a Spanish, multicentre, open-label, single arm phase II study

**DOI:** 10.1186/1471-2407-13-427

**Published:** 2013-09-20

**Authors:** Marta Martín-Richard, Bartomeu Massutí, Eva Pineda, Vicente Alonso, Maribel Marmol, Daniel Castellano, Emilio Fonseca, Antonio Galán, Marta Llanos, Maria Angeles Sala, Carlos Pericay, Fernando Rivera, Javier Sastre, Ángel Segura, Maria Quindós, Pascal Maisonobe

**Affiliations:** 1 Medical Oncology Department, Hospital de la Santa Creu y Sant Pau, Av. Sant Antoni Mª Claret, 167, Barcelona 08025, Spain; 2 Medical Oncology Department, Hospital General Universitario de Alicante, Alicante, Spain; 3 Medical Department, Ipsen Pharma, Barcelona, Spain; 4 Medical Oncology Department, University Hospital Miguel Servet, Zaragoza, Spain; 5 Medical Oncology Department, Hospital Clinic i Provincial, Barcelona, Spain; 6 Medical Oncology Department, University Hospital 12 de Octubre, Madrid, Spain; 7 Medical Oncology Department, University Hospital Clínico Salamanca, Salamanca, Spain; 8 Medical Oncology Department, Hospital de Sagunto, Sagunto, Spain; 9 Medical Oncology Department, University Hospital Canarias, Santa Cruz de Tenerife, Spain; 10 Medical Oncology Department, Hospital Universitario de Basurto, Bilbao, Spain; 11 Medical Oncology Department, Hospital Universitari de Sabadell, Corporació Sanitaria Universitaria Parc Tauli, Sabadell, Spain; 12 Medical Oncology Department, Hospital Marqués de Valdecilla, Santander, Spain; 13 Medical Oncology Department, Hospital Clínico San Carlos, Madrid, Spain; 14 Medical Oncology Department, Hospital Universitari i Politecnic La Fe, Valencia, Spain; 15 Medical Oncology, Hospital A Coruña, A Coruña, Spain; 16 Global Medical Affairs, Ipsen Pharma, Boulogne Billancourt, France

**Keywords:** Neuroendocrine tumours, Lanreotide autogel, Antiproliferative effect, Somatostatin analogues, Phase II clinical trial

## Abstract

**Background:**

Somatostatin analogues (SSAs) are indicated to relieve carcinoid syndrome but seem to have antiproliferative effects on neuroendocrine tumours (NETs). This is the first prospective study investigating tumour stabilisation with the long-acting SSA lanreotide Autogel in patients with progressive NETs.

**Methods:**

This was a multicentre, open-label, phase II trial conducted in 17 Spanish specialist centres. Patients with well-differentiated NETs and radiologically confirmed progression within the previous 6 months received lanreotide Autogel, 120 mg every 28 days over ≤92 weeks. The primary endpoint was progression-free survival (PFS). Secondary endpoints were response rate, tumour biomarkers, symptom control, quality of life (QoL), and safety. Radiographic imaging was assessed by a blinded central radiologist.

**Results:**

Of 30 patients included in the efficacy and safety analyses, 40% had midgut tumours and 27% pancreatic tumours; 63% of tumours were functioning. Median PFS time was 12.9 (95% CI: 7.9, 16.5) months, and most patients achieved disease stabilisation (89%) or partial response (4%). No deterioration in QoL was observed. Nineteen patients (63%) experienced treatment-related adverse events, most frequently diarrhoea and asthenia; only one treatment-related adverse event (aerophagia) was severe.

**Conclusion:**

Lanreotide Autogel provided effective tumour stabilisation and PFS >12 months in patients with progressive NETs ineligible for surgery or chemotherapy, with a safety profile consistent with the pharmacology of the class.

**Trial registration:**

ClinicalTrials.gov Identifier NCT00326469; EU Clinical Trial Register EudraCT no 2004-002871-18.

## Background

Somatostatin has widespread inhibitory effects on the endocrine system due to exocrine, endocrine, paracrine and autocrine actions; it also regulates cell proliferation in normal and tumour tissue, through actions mediated via five G-protein-coupled somatostatin receptors [1]. Both forms of native somatostatin (−14 and −28) have limited therapeutic viability due to extremely short half-lives (~1–3 min). Therefore, synthetic somatostatin analogues (SSAs) have been developed that provide prolonged activity while maintaining moderate- or high-affinity binding to various somatostatin receptor subtypes [2].

The goals of pharmacological treatment for neuroendocrine tumours (NETs) are to control symptoms, induce tumour regression or stabilise tumour growth, and improve survival. Currently, two long-acting SSAs (lanreotide Autogel and octreotide LAR) are available commercially for long-term management of the symptoms associated with functioning NETs, both of which are administered once a month and are generally well tolerated [3]. Treatments used for disease control include interferon and chemotherapy. Most recently, new drugs such as everolimus, an oral inhibitor of mammalian target of rapamycin (mTOR), and sunitinib, a tyrosine kinase inhibitor, have shown efficacy in controlling NETs, and in particular, pancreatic NET [4-6].

Recent data suggest that SSAs also have antiproliferative effects in NET. These effects are mediated directly, by inhibition of tumour cell proliferation and apoptosis following receptor activation, and indirectly via inhibitory effects on mitogenic growth factors (e.g. insulin growth factor-1 [IGF-1]) and tumour angiogenesis [7,8]. In clinical studies, SSAs have been shown to stabilise NETs where proliferation occurs slowly, but tumour regression is rare [9,10]. Data from a randomised phase III trial in functioning and non-functioning metastatic midgut NETs showed that octreotide LAR prolonged time to tumour progression compared with placebo [11]. While a large randomised study with lanreotide Autogel versus placebo in non-functioning NETs completed mid-2013 and results on its antiproliferative effects are due to be published in late 2013 or early 2014 [12], smaller studies with lanreotide have shown prolonged stabilisation and, in several cases, partial responses, thereby indirectly suggesting antiproliferative effects. This body of evidence has mostly comprised prospective studies of lanreotide immediate release [13-16] and microparticles [17-22]. There have also been two recent retrospective studies of the long-acting depot preparation lanreotide Autogel [23,24] and an earlier prospective study of lanreotide Autogel versus microparticles [25].

This is the first prospective study of lanreotide Autogel to evaluate tumour growth stabilisation (using blinded evaluation) and adverse effects of treatment in patients with documented progressive NET. It is also one of the longest prospective evaluations published to date of any lanreotide formulation for NET treatment.

## Methods

### Patients

Eligible patients were adults (age >18 years) with a histopathological diagnosis of advanced, well-differentiated gastroenteropancreatic, bronchopulmonary NET or neuroendocrine carcinoma (according to the World Health Organization classification [26]) and who were not candidates for chemotherapy or surgery. Other inclusion criteria were: measurable disease and disease progression in the 6 months before study inclusion (defined according to RECIST 1.0 criteria [27]); grades 0–2 on the Eastern Cooperative Oncology Group (ECOG) general status assessment scale; and positive somatostatin receptor scintigraphy. Patients were excluded if their disease was suitable for complete surgical resection, had progressed in the first 6 months after diagnosis, or if they had bowel obstruction due to a carcinoid tumour. Other exclusion criteria were: hepatic artery embolisation or radionucleotide therapy in the preceding 3 months or scheduled during the study; SSA treatment in the preceding 6 months; or radiotherapy, chemotherapy or interferon in the preceding 4 weeks or scheduled during the study; and comorbid disease that prevented understanding of and/or compliance with treatment.

### Study design and interventions

This study was a multicentre, open-label, phase II trial (with blinded central radiographic evaluation) conducted in 17 specialist centres in Spain (see Additional file 1) between May 2006 and November 2009. The protocol and amendments, patient information leaflet and informed consent document were approved by independent ethics committees at all study centres, and also by the Spanish Ministry of Health. The trial was conducted in accordance with the Declaration of Helsinki and Good Clinical Practice guidelines, and all patients provided written informed consent. The study is registered with ClinicalTrials.gov (NCT00326469) and the EU clinical trials register (2004-002871-18). Protocol amendments implemented after the study commenced are summarised in (Additional file 2: Table S1).

Lanreotide Autogel, 120 mg, was administered by deep subcutaneous injection at baseline and every 28 (±5) days thereafter by trained study personnel until 23 injections had been received over ≤92 weeks or until study withdrawal or death. Concomitant treatments were allowed at the investigators’ discretion although patients requiring additional lanreotide Autogel (other than at study visits), other SSAs, chemotherapy, interferon, radiotherapy, or surgery other than for local palliation to known lesions, were withdrawn. Patients were also withdrawn if there was disease progression, adverse events (AEs) deemed unacceptable, or a major protocol violation.

### Assessments and endpoints

Efficacy (radiological, biochemical and clinical) and pharmacokinetic (PK) assessments were performed every 12 weeks and at the final study evaluation, 28 (±5) days after the final lanreotide dose. AEs and concomitant treatments were recorded at each 4-weekly treatment visit and at the final evaluation.

The primary objective was to assess the efficacy of lanreotide Autogel on tumour growth stabilisation. The primary efficacy endpoint was progression-free survival (PFS), defined as time from study entry to tumour progression or early death, based on radiographic scans every 12 weeks (magnetic resonance imaging [MRI] or computed tomography [CT], depending on disease location and investigator choice). Radiographic imaging was assessed by an independent central radiologist who was blinded to patient identity and imaging test dates.

Secondary efficacy analyses and endpoints included: factors predictive of PFS and tumour growth control, defined as time from study entry to last assessment showing stable disease; response rate (RECIST 1.0); tumour biomarkers (chromogranin A [CgA] and urinary 5-hydroxyindole acetic acid [5-HIAA]); self-reported NET symptoms on a 3-point Likert scale; and quality of life (QoL), assessed using the European Organization for Research and Treatment of Cancer Quality of Life Questionnaire (EORTC QLQ)-C30. Metabolic biomarker (insulin, C-peptide, and gastrin) levels were also pre-determined secondary efficacy endpoints but data were collected from too few patients to provide evaluable data (insulin and C-peptide samples in one patient, gastrin samples in two). The analytical methodologies and results are thus not described further in this article.

Safety analyses included AEs (coded using the Medical Dictionary for Regulatory Activities [MedDRA] version 11.1), vital signs, and serum haematology and biochemistry. Blood samples for analysis of lanreotide trough serum levels and, if applicable, for evaluation of the presence and specificity of anti-lanreotide antibodies were collected at screening and at weeks 8, 20, 32, 44, 56, 68 and 92, just before drug administration.

Tumour biomarkers, serum lanreotide concentrations and anti-lanreotide antibodies were analysed in a central laboratory. Serum CgA levels were determined using a radioimmunoassay (RIA) with coefficients of variation (CVs) of 6–10% (Cisbio International, Gif-sur-Yvette, France), and urinary 5-HIAA levels were measured using high-performance liquid chromatography with CVs of 4–6% (Bio-rad Laboratories GmbH, München, Germany). Circulating lanreotide concentrations were measured using a validated RIA with [^125^I]-labelled lanreotide as a competitor for the quantification of lanreotide [28]. During assay validation, the lower limit of quantification for lanreotide was 0.078 ng/mL and CVs were 2.3–13.6%. Anti-lanreotide antibodies were detected using a radioimmunoprecipitation assay (RIPA) with 1-propranolol for immune-complex precipitation and [^125^I]-labelled lanreotide as the tracer. Results were expressed as the percentage of precipitation. During validation, the assay sensitivity was 1/3,200,000 using a rabbit polyclonal anti-lanreotide antibody and intra- and inter-assay precision values were <5.7% and <5.3%, respectively. The screening assay cutpoint was determined as the 95th percentile of the distribution obtained with pre-treatment samples from 22 patients from the study. Any sample with a percentage precipitation above the screening cutpoint was subjected to a confirmatory RIPA in the presence of a large amount of unlabelled lanreotide (competitor). If the competitor reduced the response by ≥30%, the serum sample was considered positive for the presence of anti-lanreotide antibodies.

### Statistical analysis

A sample size of 30 was required based on a minimum precision of 15.5% for the proportion of patients progression-free at 1 year and 2 years (assuming that 25% and 12% of patients would be progression-free at 1 year and 2 years, respectively) and an alpha error of 0.05 (bilateral). Accordingly, the estimation error (half-width of 95% confidence interval [CI]) for the proportion of patients progression-free at 1 year was planned to be 0.155 and at 2 years was 0.116.

Efficacy and safety analyses were based on data from all patients who received at least one dose of lanreotide Autogel (the intention to treat [ITT] and safety populations, respectively). Kaplan–Meier analysis was used to determine median (95% CI) PFS time (primary endpoint). Hazard ratios (HRs) (95% CI) calculated from a stepwise Cox regression model were used to determine factors predictive of PFS and tumour growth control, selecting variables with a significance level of 0.2 for entry in the model. Variables investigated comprised age, sex, tumour functionality, time from diagnosis, previous treatment, initial tumour mass, tumour origin, Ki-67 index, ECOG scale grade, serum lanreotide concentration and CgA response. The paired Student’s t-test, or Wilcoxon signed-rank test if normality assumptions were strongly violated, was used to assess mean percentage variations from baseline in biomarkers (CgA, urinary 5-HIAA) and EORTC QLQ-C30 scores. McNemar’s test was used to ascertain if variations from abnormal to normal values compared with baseline for each patient in biomarkers were due to chance or a trend towards change over time. Statistical analyses were performed with SAS version 9.1 and a 5% significance level was adopted for all tests. Descriptive statistics were used for all other endpoints.

## Results

### Patient disposition and baseline characteristics

ITT and safety populations comprised 30 patients (100% of patients screened). In all, three patients (10.0%) completed the study and 27 (90.0%) withdrew due to disease progression (n = 21 [77.8%]), safety reasons (n = 2 AEs [6.7%] and n = 1 death [3.3%]), major protocol deviation (n = 2 [6.7%]) or patient choice (n = 1 [3.3%]). Since three patients withdrew before the first disease evaluation visit (one died before the first post-baseline visit; two were subsequently deemed ineligible as one lacked a histopathological diagnosis and another lacked a positive octreotide scan), PK evaluation was based on 27 patients.

Patient demographics and clinical characteristics are presented in Table 1. Median time since diagnosis was 5.5 (range 0.2–22.2) years. Twelve of the 30 patients (40%) had midgut tumours and eight (27%) had pancreatic tumours. The majority of tumours were functioning (n = 19 [63%]), almost all of which (n = 18 [95%]) were carcinoid. Twenty-three patients (77%) had undergone surgery. In total, 10 patients (33%) had received chemotherapy and 7 (23%) interferon, at least 4 weeks before study entry; 6 (20%) had received SSAs 6 months or more prior to entering the study. Only one patient had undergone radiotherapy. Median Ki-67 index was 2.0% (range: 0–20%). Most (63%) patients presented with good performance status (i.e. ECOG grade of 0).

**Table 1:** Baseline demographic and clinical characteristics

**Characteristic**	**Patients (n = 30)**
Age, years	63.0 (40–78)
Male, n (%)	15 (50)
Time since diagnosis, years	5.5 (0.2^a^–22.2)
Prior treatment for NETs, n (%)	
Surgery	23 (76.7)
Any systemic antineoplastic therapy	15 (50.0)
Chemotherapy^b^	10 (33.3)
Interferon^b^	7 (23.3)
Somatostatin analogues^c^	6 (20.0)
Radiotherapy^b^	1 (3.3)
Origin of NETs, n (%)	
Gastroenteropancreatic NETs	
Pancreas	8 (26.7)
Stomach	1 (3.3)
Small intestine	10 (33.3)
Large intestine	3 (10.0)
Bronchopulmonary NETs	
Bronchus	4 (13.3)
Unknown	4 (13.3)
Tumour functionality, n (%)	
Functioning	19 (63.3)
Carcinoid tumour	18 (60.0)
Gastrinoma	1 (3.3)
Non-functioning	11 (36.7)
Symptomatic	9 (30.0)
Chromogranin A, μg/L	332.5 (44.1–66,056.0)
Urinary 5-HIAA, μmol/d	114.0 (19.9–1684.1)
Ki-67 index	
Ki-67 ≤2%	13 (43.3)
Ki-67 >2%	8 (26.7)
Not evaluated	9 (30.0)
Performance status: ECOG grade, n (%)	
0	19 (63.3)
1	9 (30.0)
2	2 (6.7)

Data are median (range) unless stated otherwise for quantitative parameters.

*5-HIAA*, 5-hydroxyindoleacetic acid; *ECOG*, Eastern Cooperative Oncology Group; *NET*s, neuroendocrine tumours.

^a^The patient diagnosed 0.2 years before the study (and thus apparently non-compliant with inclusion criteria) had had an earlier misdiagnosis (2 years previously) of vertebral haemangioma that should have been NET metastases.

^b^Patients who received treatment with radiotherapy, chemotherapy or interferon within 4 weeks prior to study inclusion or who were scheduled to receive it during the study were excluded from the study.

^c^Patients who received treatment with somatostatin analogues within 6 months prior to study inclusion were excluded from the study.

### Efficacy

**Figure 1:**
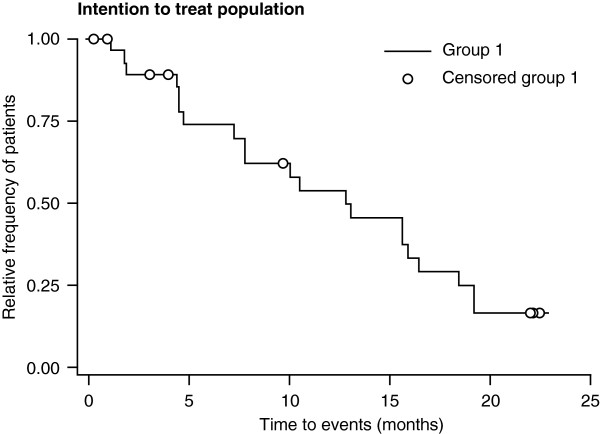
Kaplan–Meier plot of PFS among patients treated with lanreotide autogel (n = 27).

Median PFS time was 12.9 months (95% CI: 7.9, 16.5 months) (Figure 1). The PFS rate at 32 weeks (~7 months) was 69.9% (95% CI: 48.6, 83.7%), at 56 weeks (~13 months) was 49.7% (95% CI: 29.4, 67.1%), and at 80 weeks (~18 months) was 24.8% (95% CI: 10.2, 42.8%). By the last assessment at 92 weeks (~21 months), three (10%) patients were still progression free.

Ki-67 ranking of individual scores was the only predictive factor identified in the study population for either PFS or tumour growth control. Lower Ki-67 ranking predicted longer PFS (HR: 1.17, 95% CI: 1.03, 1.33; p = 0.02) and superior tumour growth control (HR 1.10, 95% CI: 0.99, 1.22; p = 0.09), although the latter was not statistically significant at the 5% level.

**Figure 2:**
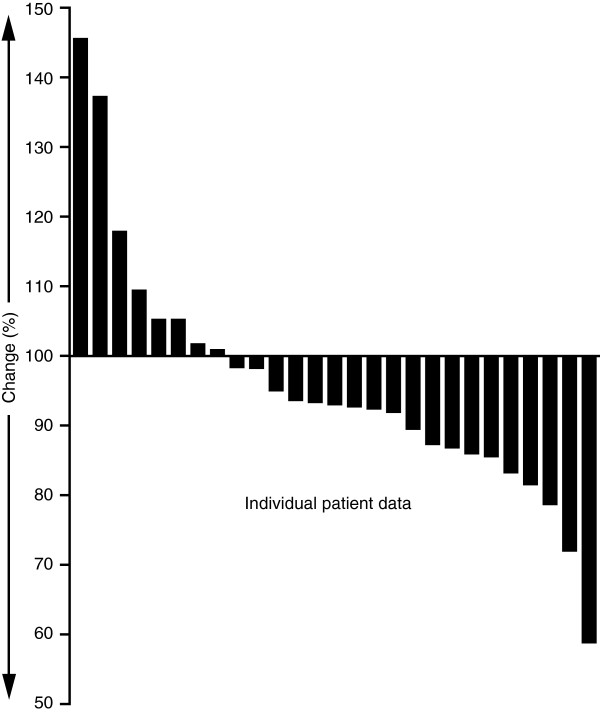
**Maximum change in sum of longest diameters of target NET lesions (n = 27)*.*** Each bar represents data for a single patient.

Changes in the sum of the longest diameter of target lesions are shown in Figure 2. None of the patients had a complete response, one (4%) had a partial response, 24 (89%) exhibited stable disease, and two (7%) experienced disease progression as their best response.

The proportion of patients in whom CgA normalised/decreased by ≥30% compared with baseline after 8 weeks was significantly greater than expected by chance (70%; 95% CI: 53.1%, 87.6%; McNemar’s test: p = 0.0002); values varied from 53% to 67% at subsequent assessments, remaining significant until week 68 (when CgA was assessed for only 6 patients). The median decrease from baseline in CgA concentration was significant after 8 weeks (−35%; range, –91.8 to −7.8%; Wilcoxon’s test: p < 0.001); median decreases varied from −31% to −17% at subsequent assessments, although not all reductions reached statistical significance. After 8 weeks, in the 19 patients with functioning tumours, the median decrease from baseline in urinary 5-HIAA was −30% (range: –90.4, 24.7%; paired Student’s t-test: p = 0.0006); median change varied within −64% to −29% at subsequent assessments but changes were not all statistically significant.

Of the 19 patients with tumours classified as functioning based on amine and/or peptide secretions, nine (47%) had symptoms related to NET at baseline and two (11%) developed symptoms after starting treatment. All symptomatic patients reported diarrhoea, one also experienced asthenia and another reported shortness of breath and tachycardia; no symptoms were severe. Of the nine patients who were symptomatic at baseline, five (56%) achieved complete symptom relief after starting treatment and three (33%) developed new symptoms during treatment. One patient had no recorded symptom evaluation.

EORTC QLQ-C30 scores were generally stable from baseline to week 56 and showed a non-significant trend towards improvement across the functional multi-item scales and the global QoL multi-item scale. The multi-item symptom scores and single-item scales for adverse effects showed some fluctuation but were generally stable over the same period (Additional files 3 and 4: Figures S1a and S1b). Completed questionnaires were available for fewer than 10 patients after 56 weeks.

### Safety and tolerability

Median exposure to study medication was 291 days (range: 1–702 days). Twenty-five of the 30 patients (83%) experienced at least one AE; 63% of patients had treatment-related AEs (Table 2). The most common treatment-related AEs were diarrhoea (n = 12 [40%]), asthenia (n = 6 [20%]), flatulence (n = 3 [10%]) and injection-site pain (n = 3 [10%]). Only one of these AEs was severe (aerophagia), and another was serious (acute renal failure) but resolved without sequelae. Three patients (11%) withdrew due to AEs: these were the aforementioned serious AE (acute renal failure), a non-serious AE unrelated to treatment (carcinoid syndrome), and a serious AE unrelated to treatment (gallbladder fistula; patient later died). Two patients (7%) died during the study period; neither death was treatment-related and both were secondary to disease progression (one due to intestinal obstruction and one due to gallbladder fistula).

**Table 2:** Patients reporting mild, moderate or severe treatment-related adverse events during treatment with lanreotide autogel

**Organ system**	**Severe**	**Moderate**	**Mild**	**Total**
Any	1 (3.3)	8 (26.7)	17 (56.7)	19 (63.3)
Gastrointestinal	1 (3.3)	4 (13.3)	12 (40.0)	13 (43.3)
General and	0	5 (16.7)	9 (30.0)	10 (33.3)
injection site				
Neurological	0	0	3 (10.0)	3 (10.0)
Metabolic and nutritional	0	1 (3.3)	1 (3.3)	2 (6.7)
Infections	0	1 (3.3)	0	1 (3.3)
Skin and subcutaneous tissue	0	0	1 (3.3)	1 (3.3)
Ear and labyrinth	0	0	1 (3.3)	1 (3.3)
Renal/urinary tract	0	1 (3.3)^a^	0	1 (3.3)
Reproductive system and breast	0	0	1 (3.3)	1 (3.3)
Vascular	0	0	1 (3.3)	1 (3.3)

Data are number (%) of patients.

^a^Event was serious AE (acute renal failure) but resolved without sequelae.

### Pharmacokinetics and immunogenicity

Steady-state levels of lanreotide (5.2 ± 2.0 ng/mL) were reached at week 20 after five injections. Mean trough lanreotide levels were broadly stable reaching a maximum of 6.0 ± 2.9 ng/mL at week 32 (Additional file 5: Figure S2). After steady-state levels were achieved, lanreotide serum concentrations were maintained throughout the 92-week treatment period (mean: 5.3 ± 2.0 ng/mL at week 92). At week 8, serum from all 25 patients tested was negative for anti-lanreotide antibodies; at subsequent assessments, anti-lanreotide antibodies were detected in two patients (7%), one of them temporarily.

## Discussion

The current study provides new evidence for the antiproliferative effect of long-acting lanreotide Autogel in NET. In patients with radiologically demonstrated progressive disease in the previous 6 months, we showed that lanreotide Autogel, 120 mg every 28 days, was associated with a median PFS of more than 12 months as determined by blinded central evaluation. Ki-67 was the only factor predictive of PFS – such that a lower Ki-67 predicted longer PFS – and there was no deterioration in QoL during the study. Treatment was generally well tolerated, with a safety profile consistent with the pharmacology of the drug.

There is indirect evidence that lanreotide may have antiproliferative effects on NET. Prospective nonrandomised studies have shown tumour responses or long periods of tumour stabilisation with the immediate-release [14-16] and microparticle formulations [17-22]. There have also been reports of tumour growth control in two long-term retrospective studies of the long-acting depot (Autogel) formulation [23,24] and in a randomised study of lanreotide microparticles versus Autogel over 18 weeks [25]. Further evidence showed similar tumour stabilisation with lanreotide immediate-release, interferon alpha or lanreotide plus interferon alpha in patients with progressive NET over 12 months [13]. This offers stronger support for an antiproliferative effect but lacks a placebo arm for comparison.

Direct support for an antiproliferative effect in NET has been reported previously for octreotide LAR, the other commercially available long-acting SSA [11]. In this randomised, double-blind trial of patients with non-functioning midgut NETs (the PROMID study), the time to tumour progression was significantly longer in patients receiving octreotide LAR than in those receiving placebo (14 and 6 months, respectively) when hepatic tumour burden was ≤10%. Although between-study comparisons should be made with caution, PFS and disease stabilisation were similar in PROMID and the current study despite key differences in study design. In our study, for example, patients had more advanced disease, had previously received systemic treatments, and had pancreatic, intestinal or lung NETs, while in PROMID the population was limited to those with midgut NETs. A particular strength of our study is that all participants had documented progressive disease within the previous 6 months whereas the proportion with disease progression at enrollment is not reported for PROMID. On the other hand, PROMID was a placebo-controlled study, while ours was not. Data from two large, ongoing, randomised, double-blind, placebo-controlled studies with lanreotide Autogel are thus awaited with interest. The first was conducted in a homogeneous population of patients with non-functioning gastroenteropancreatic NETs (CLARINET study: NCT00353496) [12] and the second in patients with a history of carcinoid syndrome (ELECT study: NCT00774930) [29]. Data from these studies are expected late 2013 or early 2014.

Other treatment modalities that have shown promise as antiproliferative agents for advanced NET include mTOR or tyrosine kinase inhibitors. Recent clinical trials have demonstrated that these molecular targeted therapies can provide tumour stabilisation in patients with advanced pancreatic NET (PFS of 11.0 months for everolimus and 11.4 months for sunitinib) [30,31]. The combination of everolimus and octreotide LAR also stabilised tumour growth in patients with carcinoid NET [32]. To date, some preliminary research with octreotide and lanreotide in various NET types has suggested that combination therapy with molecular targeted therapies may provide antiproliferative effects that take advantage of potential synergies between these agents’ different modes of action [4-6].

No unexpected safety signals were noted, which is consistent with other recent lanreotide studies in patients with NET [23,24]. The most common AEs were primarily gastrointestinal and asthenia, and the AEs observed did not lead to a higher than anticipated incidence of treatment-related withdrawal.

Lanreotide trough serum concentrations remained stable for the duration of this study suggesting sustained exposure to lanreotide Autogel for at least 92 weeks. Lanreotide treatment also exhibited limited immunogenicity, as only two patients developed antibodies (in one case, temporarily). Therefore, there is low risk that antibodies might adversely affect efficacy, safety or pharmacokinetics.

This study has several limitations. First, as noted earlier, it was a single-arm (and thus unblinded) study in a relatively small population of patients with functioning or non-functioning progressive NETs of different origins. While the finding of substantial antiproliferative efficacy is promising, data from the CLARINET study are expected to corroborate findings. Second, the study was not powered sufficiently to assess potential predictive factors, nor did it assess hepatic involvement and its effect on PFS. Third, the exclusion from our study of patients with disease progression in the 6 months following diagnosis likely excluded those with more aggressive disease and could have biased the results towards longer PFS. However, in the RADIANT 1–3 studies [30,32,33], where such patients were not excluded, those whose tumors progressed within 6 months of diagnosis represented <4%, <14% and <9% of the respective study populations. It is therefore unlikely that more than a few cases of more aggressive disease were excluded from our study and, as such, it is unlikely that this affected the results. Finally, a full PK profile was not assessed but will be determined from the phase III studies of lanreotide and used in population PK studies.

## Conclusions

These findings show that lanreotide Autogel achieved clinically meaningful PFS (>12 months) in patients with radiologically confirmed progressive, well-differentiated NETs, strongly supporting an antiproliferative effect. Lanreotide also provided symptom control with stable QoL, and a favourable tolerability profile. These findings are encouraging, particularly in this group of patients with limited treatment options. In addition to future clinical trials of lanreotide Autogel for NET stabilisation, further research on treatment strategies that combine lanreotide and molecular targeted therapies will help characterise the clinical utility of lanreotide-based combinations in the management of NETs.

## Abbreviations

AE: Adverse event; CLARINET: Controlled study of lanreotide antiproliferative response in NET; CT: Computed tomography; CgA: Chromogranin A; ECOG: Eastern cooperative oncology group; EORTC QLQ-C30: European organization for research and rreatment of cancer quality of life questionnaire C30; 5-HIAA: 5-hydroxyindole acetic acid; IGF-1: Insulin growth factor-1; MedDRA: Medical dictionary for regulatory activities; MRI: Magnetic resonance imaging; mTOR: Mammalian target of rapamycin; NET: Neuroendocrine tumours; PFS: Progression-free survival; PK: Pharmacokinetic; QoL: Quality of life; PROMID: Placebo-controlled, double-blind, prospective, Randomized study on the effect of Octreotide LAR in the control of tumor growth in patients with metastatic neuroendocrine MIDgut tumors; RECIST: Response evaluation criteria in solid tumours; SSA: Somatostatin analogue.

## Competing interests

EP and PM are employees of Ipsen. All remaining authors declare that they have no competing interests.

## Authors’ contributions

MMR, BM, and EP contributed to study concept and design. MMR, BM, EP, VA, DC, EF, and PM participated in data analysis and interpretation. MMR, BM, VA, MM, DC, EF, AG, ML, MAS, CP, FR, JS, AS, and MQ were involved in data acquisition. All authors were involved in preparation of the manuscript. All authors read and approved the final manuscript.

## Pre-publication history

The pre-publication history for this paper can be accessed here:

http://www.biomedcentral.com/1471-2407/13/427/prepub

## Supplementary Material

Additional file 1 List of study centres. Click here for file

Additional file 2: Table S1 Protocol changes occurring during the study. Click here for file

Additional file 3: Figure S1a Mean scores on the EORTC QLQ-C30 scale (n = 27) during treatment with lanreotide Autogel. Panel A: functional domains. Click here for file

Additional file 4: Figure S1b Mean scores on the EORTC QLQ-C30 scale (n = 27) during treatment with lanreotide Autogel. Panel B: symptoms. Click here for file

Additional file 5: Figure S2 Mean (SD) serum lanreotide concentration (ng/mL) over time in patients receiving lanreotide Autogel (n = 27). Click here for file
