# Supplementary material for: Antiproliferative effects of lanreotide autogel in patients with progressive, well-differentiated neuroendocrine tumours: a Spanish, multicentre, open-label, single arm phase II study
Source: BMC Cancer. 2013 Sep 20;13:427. doi: 10.1186/1471-2407-13-427 (PMC3853091; doi:10.1186/1471-2407-13-427)
Supplement: Additional file 1 — List of study centres. [file 1471-2407-13-427-S1.docx]

**Study centres**

*Alicante*: Hospital General, Universitario de Alicante; Hospital “Virgen de los Lirios”

*Barcelona:* Hospital Santa Creu i Sant Pau; Hospital Clínic i Provincial; Corporación Hospital Parc Taulí

*Coruña*: Hospital “Juan Canalejo”

*Madrid*: Hospital “12 de Octubre”; Hospital “Ramón y Cajal”; Hospital Clínico Universitario “San Carlos”; Hospital de la Princesa

*Salamanca*: Hospital Clínico de Salamanca

*Santander:* Hospital “Marqués de Valdecilla

*Tenerife*: Hospital Universitario de Canarias

*Valencia*: Hospital de Sagunto; Hospital “La Fe”

*Vizcaya:* Hospital de Basurto

*Zaragoza*: Hospital “Miguel Servet”
