# Supplementary material for: Antiproliferative effects of lanreotide autogel in patients with progressive, well-differentiated neuroendocrine tumours: a Spanish, multicentre, open-label, single arm phase II study
Source: BMC Cancer. 2013 Sep 20;13:427. doi: 10.1186/1471-2407-13-427 (PMC3853091; doi:10.1186/1471-2407-13-427)
Supplement: Additional file 2: Table S1 — Protocol changes occurring during the study. [file 1471-2407-13-427-S2.docx]

**Table S1. Protocol changes occurring during the study**

A number of changes were made to the protocol definitions to comply with International Conference on Harmonisation (ICH) guidelines [1]. Key changes are listed below.

| **Parameter** | **Original protocol definition** | **Definition in final analysis plan** |
| --- | --- | --- |
| Definition of ITT population | Patients who have received at least one dose of lanreotide Autogel, and who have at least one evaluation of the primary efficacy parameter | Patients who have received at least one dose of lanreotide Autogel |
| Definition of PP population | Patients who, with no major protocol violations/deviations, have received at least one dose of lanreotide Autogel, and who have at least one evaluation of the primary efficacy parameter | Patients who, with no major protocol infringements, have received at least one dose of lanreotide Autogel |
| Primary efficacy endpoint | Time to disease progression according to RECIST criteria | Efficacy of lanreotide Autogel in tumor growth stabilisation in patients with progressive disease |
| Secondary efficacy endpoints | • Includes number and location of metastases and ploidy degree. | • Excludes number and location of metastases and ploidy degree. |
|  | • Ki-67 not listed as ranked data | • Ki-67 index listed as ranked data. |
|  | • States that ‘biochemical data and response to treatment’ will be measured |  |
|  |  | • Biochemical data specified as CgA response (normalisation and/or decrease of 30% in CgA at first evaluation visit) |
|  | Quality of life determined using EORTC QLQ-C30 |  |
|  |  | • Evaluation of effects of treatment on quality of life including change from baseline in QLQ-C30 subscales (e.g. physical, role, cognitive, etc.) |
|  |  | • Evolution of sum of the longest raw diameter of target lesions |

1. International Conference on Harmonisation (ICH) Topic E9: Statistical Principles for

Clinical Trials, issued as CPMP/ICH/363/93. September 1998.

CgA, chromogranin A; EORTC QLQ-C30, European Organization for Research and Treatment of Cancer Quality of Life Questionnaire C30; ITT, intention to treat; PP, per protocol; RECIST, Response Evaluation Criteria in Solid Tumours.
